# Ventral premotor to primary motor cortical interactions during object-driven grasp in humans

**DOI:** 10.1016/j.cortex.2009.02.011

**Published:** 2009-10

**Authors:** Marco Davare, Karli Montague, Etienne Olivier, John C. Rothwell, Roger N. Lemon

**Affiliations:** a Sobell Department of Motor Neuroscience and Movement Disorders, Institute of Neurology, UCL, Queen Square, London, UK; b Laboratory of Neurophysiology, Institute of Neuroscience (INES), Université Catholique de Louvain, Brussels, Belgium

**Keywords:** Functional connectivity, EMG, Motor control, Hand shaping, Corticospinal

## Abstract

Interactions between the ventral premotor (PMv) and the primary motor cortex (M1) are crucial for transforming an object's geometrical properties, such as its size and shape, into a motor command suitable for grasp of the object. Recently, we showed that PMv interacts with M1 in a specific fashion, depending on the hand posture. However, the functional connectivity between PMv and M1 during the preparation of an actual grasp is still unknown.

To address this issue, PMv–M1 interactions were tested while subjects were preparing to grasp different visible objects requiring either a precision grip or a whole hand grasp. A conditioning–test transcranial magnetic stimulation (TMS) paradigm was used: a test stimulus was applied over M1 either in isolation or after a conditioning stimulus delivered, at different delays, over the ipsilateral PMv. Motor evoked potentials (MEPs) were recorded in the first dorsal interosseus and abductor digiti minimi muscles, which show highly differentiated activity according to grasp.

While subjects prepared to grasp, delivering a conditioning PMv pulse 6 or 8 msec before a test pulse over M1 strikingly facilitated MEPs in the specific muscles that were used in the upcoming grasp. This degree of facilitation correlated with the amount of muscle activity used later in the trial to grasp the objects.

The present results demonstrate that, during grasp preparation, the PMv–M1 interactions are muscle-specific. PMv appears to process the object geometrical properties relevant for the upcoming grasp, and transmits this information to M1, which in turn generates a motor command appropriate for the grasp. We also reveal that the grasp-specific facilitation resulting from PMv–M1 interactions is differently related to the upcoming grasp muscle activity than is that from paired-pulse stimulation over M1, suggesting that these two TMS paradigms assess the excitability of cortico-cortical pathways devoted to the control of grasp at two different levels.

## 1 Introduction

An important prerequisite for secure grasp of an object is to adopt a hand shaping congruent with its geometrical properties (Jeannerod et al., 1995). Functional imaging and electrophysiological studies have shown that the ventral premotor cortex (PMv) is activated while subjects perform grasping movements (Binkofski et al., 1999; Ehrsson et al., 2001; Grezes et al., 2003) and contains neurons that are selective for a particular grasp configuration (Murata et al., 1997; Umilta et al., 2007). A causal relationship between this PMv activation and its role in controlling hand shaping was further indicated by studying the consequences of inactivating this area using either muscimol in monkeys (Fogassi et al., 2001) or transcranial magnetic stimulation (TMS) in humans (Davare et al., 2006). Although these data indicate the importance of the close interaction between PMv and primary motor cortex (M1) in hand shaping, they do not reveal how information flows through the PMv–M1 network, which is crucial to the understanding of the visuomotor transformations that take place in this circuit.

Because corticospinal (CS) projections from PMv are very few in number and particularly sparse to the cervical enlargement where motoneurons innervating hand muscle are located (He et al., 1993), it is more probable that PMv influences hand muscles through the PMv–M1 cortico-cortical connections (Shimazu et al., 2004; Cerri et al., 2003). Electrical excitation of PMv, while itself yielding little or no effect on CS outputs to hand muscles, can exert powerful facilitation of CS outputs from M1, a facilitation that appears to be largely relayed through M1 (Schmidlin et al., 2008) and to be specific for particular object-grasp configurations (Prabhu et al., 2009). Recently, by means of a conditioning–test (C–T) TMS paradigm, we investigated the functional connectivity between PMv and M1 while subjects were adopting different hand postures (Davare et al., 2008). We found that the resting state net inhibition from PMv to M1 is converted into a net facilitation specifically while subjects adopted a precision grip (PG) steady posture. This indicates that PMv could modulate the M1 outputs depending on the type of grasp being prepared. However, the nature of any interactions between PMv and M1 for grasp is still unknown.

To address this issue, we used a C–T TMS paradigm while subjects were asked to grasp different objects using either an index-thumb opposition (PG) or a whole hand grasp (WHG). Specific PMv–M1 interactions were compared with the action of general cortico-cortical inputs to M1 by using a paired-pulse stimulation over M1 (M1_PP_), in which the conditioning stimulus is delivered after the test pulse. This latter technique, by revealing task-related changes of the later I-wave components (Cattaneo et al., 2005), allows us to assess the effects of general cortico-cortical inputs to M1, including not only those from PMv but also from other cortical motor areas. This comparison should shed some light on how grasp-related information is transmitted from PMv to M1 and then processed by the M1 intrinsic circuitry in order to generate an appropriate motor command for grasp.

## 2 Methods

### 2.1 Subjects

Eleven right-handed (Oldfield, 1971) volunteers (20–30 years) participated after providing informed consent. None reported neurological impairments and all were screened for adverse reactions to TMS (Keel et al., 2001). The experimental procedure was approved by the ethics committee of University College London.

### 2.2 Experimental task

**Fig. 1:**
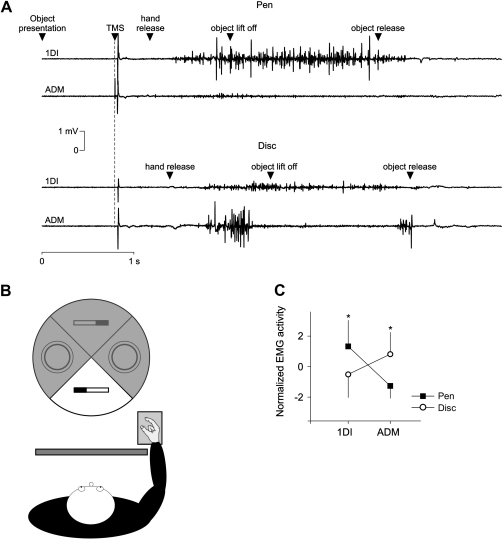
Experimental setup and muscle activity. A. Typical traces showing the event sequence during a trial where subjects had to grasp a pen (top traces) or a disc (bottom traces). TMS occurred 800 msec after object presentation and was the cue to grasp the object. B. Schematic view of the experimental setup. The hand-pad was located at 30 cm from the table edge. The turntable randomly presented the objects 30 cm ahead from the hand-pad. A screen, made from switchable transparent glass, was positioned between the subject and the turntable to allow us to control precisely the timing of object presentation. C. *Z*-score normalized EMG activity when subjects grasped either the pen or the disc. The 1DI was more active when grasping the pen compared to the disc and conversely for the ADM.

The experiment aimed at determining the functional connectivity between PMv and M1 while subjects were preparing to grasp two different objects. Participants were comfortably seated in front of a table with the right hand resting on a hand-pad located at 30 cm from the edge of the table. Subjects were asked to grasp objects at their own pace using either a PG between the index and thumb or a WHG. The two objects were a 2 cm diameter pen grasped with a PG and an 11 cm diameter disc (3 cm thickness) grasped with a WHG. The two objects had the same mass (50 g). The objects were placed 30 cm ahead of the hand-pad and were presented in a random order by means of a motorised turntable connected to a CED 1401 (Cambrigde Electonic Design, Cambridge, UK) (see Fig. 1B). The duration of visible presentation of the object was controlled by a screen (switchable transparent glass, All Brilliant Tech, Beijing) placed between the subject and the turntable. Subjects could see the object to grasp only after the screen was made transparent. A TMS pulse (see below), occurring 800 msec after object presentation (Prabhu et al., 2007), was the cue to release the hand-pad and grasp the object appropriately (Fig. 1A). Subjects had then to lift it to approximately 10 cm height and replace it on the turntable after a beep. When subjects repositioned the right hand on the hand-pad, the screen turned opaque and the next trial started after a randomised inter-trial time (7–10 sec).

### 2.3 Transcranial magnetic stimulation

To investigate PMv–M1 interactions in the left hemisphere, we used two custom-made figure-of-eight coils (7 cm outer diameter) connected to two single-pulse monophasic Magstim model 200 stimulators (Magstim Company, Whitland, UK). The conditioning (C) stimulus was delivered over PMv (see Supplemental Methods online for Stimulation sites), with anterior to posterior induced current, through a coil held tangentially to the skull with the handle pointing forward; the test (T) stimulus was delivered over M1 (see Supplemental Methods online for Stimulation sites), with posterior to anterior induced current, through a coil held perpendicularly to the central sulcus with the handle pointing backwards. The C and T stimuli were set, respectively, at 80% and 120% of the resting motor threshold (rMT) (Civardi et al., 2001), defined as the minimum intensity that induced motor evoked potentials (MEPs) ≥ 50 μV peak-to-peak in both the first dorsal interosseus (1DI) and abductor digiti minimi (ADM) in 5 out of 10 trials (Rossini et al., 1994). The rMT was determined by using a coil connected to a single-pulse Magstim stimulator and equalled on average 44 ± 5% of the maximal stimulator output (mean ± SD, *n* = 11).

In a control condition (M1–M1_C–T_ condition), both the C and T stimuli were applied over M1, with posterior to anterior induced current, through the same coil connected to two single-pulse monophasic Magstim model 200 stimulators through a Y-shaped cable (Magstim Company, Whitland, UK). This M1–M1_C–T_ condition was introduced to control for a possible spread of TMS current from PMv to M1. The coil was held tangential to the skull with the handle perpendicular to the central sulcus.

Finally, a third condition consisted of delivering paired-pulse TMS over M1 (M1_PP_). The first stimulus (120% rMT) was followed 2.5 msec later by a second stimulus (80% rMT). This procedure was used to reveal the effect of general cortico-cortical inputs to M1 during grasp preparation (Cattaneo et al., 2005; Prabhu et al., 2007). It is known that a single TMS pulse delivered to M1 produces a direct (D) wave followed by indirect (I) waves (Day et al., 1989; Rothwell, 1991). Later I-waves can reflect activity in cortico-cortical pathways to M1 (Porter and Lemon, 1993; Shimazu et al., 2004), which can be probed by using a paired-pulse TMS paradigm. The first (supra-threshold) stimulus is followed by a second (sub-threshold) stimulus over M1, delivered with an interval of 2.5 msec so as to coincide with I-wave activity generated by the first pulse (Cattaneo et al., 2005; Prabhu et al., 2007; Ziemann et al., 1998).

### 2.4 Experimental procedure

Subjects had to perform 8 blocks of 48 trials, 4 in the PMv–M1 condition and 4 M1–M1_C–T_ in the condition. The C–T interval (inter-stimulus interval, ISI) was varied randomly between 1, 2, 4, 6, 8, 10 and 15 msec. T alone was delivered in 1 out of 8 trials and the MEP amplitudes measured in this condition were used as baseline values. Altogether, for either PMv–M1 or M1–M1 condition, 192 trials were performed: 12 trials for each ISI (8 conditions: 7 C–T intervals + T alone) and for each object (pen and disc).

In the M1_PP_ experiment, 2 blocks of 40 trials were carried out. In half of the trials, paired-pulse stimuli were delivered (ISI 2.5 msec). In the other half, a single test stimulus was delivered and these MEPs were used as baseline values. For both TMS conditions (paired-pulse 2.5 msec and T alone), the object was either a pen or a disc.

### 2.5 Data acquisition and analysis

The Magstim stimulators were triggered using Spike2 software and CED data acquisition interface (Cambridge Electronic Design, Cambridge, UK). EMG activity was recorded with bipolar surface electrodes (belly-tendon), one pair positioned over the 1DI and the other over ADM. The raw EMG signals were amplified (Neurolog, Digitimer Ltd, UK) and digitized at 5 kHz for offline analysis.

The reaction time (RT) was defined as the delay between the TMS pulse (C stimulus) and the hand-pad release. The movement duration (MD) was computed as the delay between the hand-pad release and object lift off, detected by means of an optical sensor placed just below the turntable. The peak-to-peak amplitude of each individual MEP was measured and expressed as a percentage of the control (baseline) MEP (T stimulus alone) gathered during the same block. Trials where any EMG activity was present during the movement preparation period (800 msec) were discarded. The muscle activity involved in the preshaping of the hand during either the PG or WHG was estimated by computing the area-under-curve of the rectified EMG between the time at which subjects left the hand-pad and 100 msec before the object lift off. For each muscle and each subject, the EMG values were *Z*-score normalized to the grand average of each subject (both grasps).

### 2.6 Statistical analyses

For each site of C stimulus delivery (M1 or PMv), repeated measure ANOVAs (ANOVA_RM_) were performed on the RT, MD, relative MEP amplitudes and EMG values with C–T interval (1, 2, 4, 6, 8, 10, 15 msec or T alone) and grasp (PG or WHG) as within subject factors. Planned post-hoc comparisons (each C–T interval with respect to T alone) were performed using Dunnett's test. Correlations between the amount of MEP facilitation and muscle activity during the preshaping of the hand were performed using the Pearson procedure.

## 3 Results

The two objects used in this study, a pen and a disc, required a different and well-defined pattern of muscle activity during hand preshaping for grasp (Fig. 1A and C). Grasping the pen required significantly greater activity in the 1DI, agonist of the PG, compared with grasping the disc. In contrast, when the disc had to be grasped, the ADM, agonist of the little finger abduction, was significantly more active than when grasping the pen (ANOVA, grasp main effect, *F* = 15.85, *p* < .001, Fig. 1C). The RT and MD were 873.4 ± 106.4 and 1421.5 ± 246.8 msec, respectively. None of the TMS conditions, C–T intervals nor grasp affected the RT and MD (ANOVA main effect of interval or grasp: both *F* < 1).

**Fig. 2:**
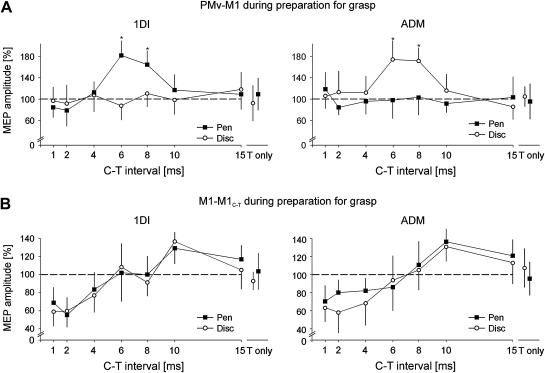
PMv–M1 and M1–M1 interactions during preparation for grasp. A. Relative amplitude of MEPs recorded from the 1DI and ADM while subjects were preparing to grasp either the pen or the disc. Values on the *Y*-axis represent the relative MEP amplitudes resulting from a supra-threshold test (T) stimulus applied over M1 preceded by a sub-threshold conditioning (C) stimulus applied over PMv at different intervals (*X*-axis). T-only values represent the baseline MEP amplitude, i.e., when no conditioning pulse was delivered. B. Same as A but the conditioning pulse was delivered over M1. The error bars show 1 SD.

During the preparation of grasp, 800 msec after objects were visually presented to the subjects, PMv–M1 interactions were tested by means of a conditioning–test TMS paradigm. We aimed at determining whether delivering a conditioning pulse over PMv could differentially modulate the M1 outputs during preparation of either the PG or WHG grasps. We found that delivering the C pulse over PMv 6 or 8 msec before the T pulse over M1 strikingly facilitated the MEP recorded in the 1DI only when preparing the PG (ANOVA, interval × grasp interaction: *F* = 6.43, *p* = .005; post-hoc: both *p* < .002, Fig. 2A). Similarly, applying the 6 and 8 msec C–T intervals facilitated the MEP recorded in the ADM, but only when preparing the WHG (ANOVA, interval × grasp interaction, *F* = 5.75, *p* = .012; post-hoc: both *p* < .027, Fig. 2A). No significant effect of the C pulse was found at other intervals (post-hoc: all *p* > .05). These results indicate that delivering a conditioning pulse over PMv induces a muscle-specific facilitation of the M1 outputs, depending on the upcoming grasp. We also examined, across subjects, the difference in EMG activity for the grasp themselves and whether this was correlated with the difference in the MEP recorded during movement preparation (MEP pen *vs* MEP disc, average of 6 and 8 msec ISI). We found that, across subjects, the difference of EMG activity (EMG pen *vs* EMG disc) correlated with the difference in the MEP amplitude (1D1: *r* = .79, *p* = .003; ADM: *r* = .78, *p* = .001; Fig. 4A). This result indicates that specific changes in the excitability of M1 outputs to hand muscles, induced by PMv stimulation, are directly correlated to the different muscle pattern used later to grasp the two objects.

The M1–M1_C–T_ condition was introduced to control for a possible non-specific direct spread of current from the PMv conditioning stimulus towards M1, rather than through its connections with M1. The rationale is that if the conditioning pulse over M1 has a different effect that conditioning PMv, then unspecific spread from PMv to M1 is unlikely to have occurred. Indeed, in the M1–M1_C–T_ condition, we corroborated results of previous studies (Kujirai et al., 1993), namely a decrease in MEP amplitude in both 1DI and ADM muscles for short intervals (1, 2, 4 msec, short interval cortical inhibition or SICI) and an increase for longer ones (10 msec, short intracortical facilitation or SICF) (ANOVA main effect of interval, both *F* > 3.47, both *p* < .017; post-hoc: all *p* < .003, Fig. 2B); conditioning stimuli delivered over M1 6, 8 or 15 msec before the test shock had no effect on MEP amplitude (both *p* > .05). Interestingly, SICI and SICF in both muscles did not change depending on the grasp (pen or disc) (ANOVA interval × grasp interaction: *F* = .86, *p* > .05). This indicates that the PMv–M1 selective facilitatory effect we observed in the muscle agonist of the upcoming grasp was not due to a downstream change in the M1 intracortical circuit per se, but rather to a modulation in the PMv–M1 functional connectivity.

In order to compare the PMv–M1 interactions with other cortico-cortical inputs to M1, we conducted an additional paired-pulse experiment over M1 (M1_PP_). It is known that excitability changes in these inputs can be revealed by using a paired-pulse TMS paradigm in which the first (supra-threshold) stimulus is followed by a second (sub-threshold stimulus) over M1, delivered with an interval (2.5 msec) which coincides with the I-wave activity generated by the first pulse (see Methods; Cattaneo et al., 2005; Prabhu et al., 2007).

**Fig. 3:**
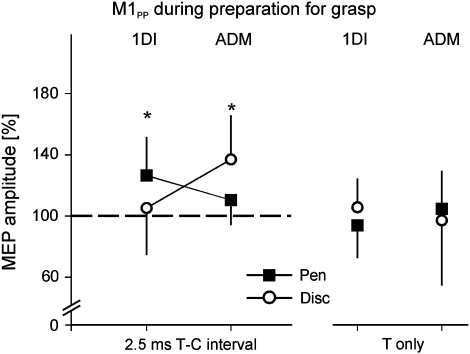
M1 cortico-cortical inputs during preparation for grasp. Values on the *Y*-axis represent the relative MEP amplitude resulting from delivering the conditioning pulse over M1 2.5 msec after the test pulse. The error bars show 1 SD.

**Fig. 4:**
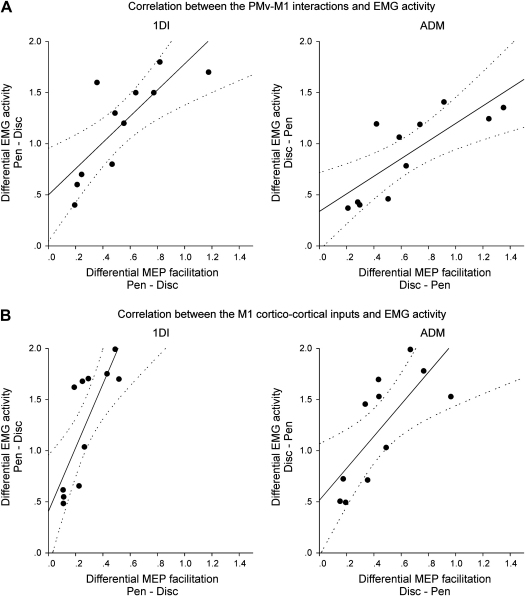
Amount of MEP facilitation related to the pattern of grasp muscle activity across subjects. Correlations between the differential MEP facilitation and the muscle activity during the preshaping of the hand. Values of MEP facilitation gathered from C–T intervals of 6 and 8 msec (mean amount of facilitation in each subject at the two C–T intervals, A) or from the 2.5 msec M1_PP_ paradigm (B). The *X*-axis shows the difference between the MEP amplitude recorded while subjects (*n* = 11) prepared grasp of either the disc or the pen (MEP pen – MEP disc for 1DI or MEP disc – MEP pen for ADM). The *Y*-axis represents the difference in EMG activity between the two objects (EMG pen – EMG disc for 1DI or EMG disc – EMG pen for ADM). Note that the slope of the regression line is steeper for MEPs acquired during the M1_PP_ condition.

Similarly to the PMv–M1 condition, we found that delivering paired-pulse TMS over M1 increased the MEP amplitude during movement preparation only in the muscle that was agonist of the upcoming grasp (ANOVA interval × grasp interaction: both *F* > 5.83, both *p* < .039, Fig. 3). However, the selective facilitation observed in this M1_PP_ condition was smaller than the facilitation we found in the PMv–M1 paradigm (paired *t*-test: all *p* < .037). In line with this result, we found that the difference in the MEP facilitation (MEP pen *vs* MEP disc, ISI 2.5 msec) was still proportional to the difference in the muscle activity during the upcoming grasp (EMG pen *vs* EMG disc) (1DI: *r* = .71, *p* = .008; ADM: *r* = .68, *p* = .011, Fig. 4B). However, when performing a linear regression between the MEP difference and the EMG difference (see Fig. 4), we found that the regression slope was steeper for the M1_PP_ condition compared to the condition PMv–M1 (mean slope for M1_PP_: 2.78; PMv–M1: 1.24; paired *t*-test: *p* = .021). Although it is difficult to determine whether this difference in slope is actually related to a distinct processing of the grasp-related information by PMv–M1 interactions or by intrinsic M1 circuitry, it does suggest that the two TMS paradigms used here (PMv–M1 or M1_PP_) could probe different elements of this network and hence, assess the transformation of grasp-related visuomotor information at two different levels.

## 4 Discussion

When subjects grasp different objects using a precision grip or a whole hand grasp, the activity in the hand muscles shows a characteristic pattern for each of the grasps. The 1DI muscle is more active when grasping the pen than the disc and, conversely, the ADM shows greater activity for the disc compared with the pen. The present study demonstrates that, during grasp preparation, interactions between PMv and M1 are modulated depending on the object to be grasped. If the pen was presented to the subjects, the PMv–M1 interactions showed a specific facilitation of 1DI, the muscle that acts as a prime mover for the PG. In contrast, when the disc was the object to grasp, there was an enhancement of the CS output of the ADM, the muscle more active during spreading of the digits for WHG. The grasp-specific facilitation originating from PMv was found to be greater than the facilitation obtained by testing the M1 cortico-cortical inputs and always matched the muscle pattern used in the upcoming grasp. This suggests that the processing of grasp-related information by PMv–M1 interactions prior to the generation of a motor command appropriate for the upcoming grasp is rather different to that in the other cortico-cortical inputs probed by paired-pulse TMS over M1 itself.

We found that delivering a sub-threshold conditioning pulse over PMv 6 or 8 msec before a supra-threshold test stimulus over M1 specifically facilitates the MEP in the muscle that will be used in the upcoming grasp. Electrophysiological studies in monkeys have shown that PMv contains neurons that discharge specifically for a particular object or grasp (Murata et al., 1997; Umilta et al., 2007). Thus, PMv comprises different cell populations, constituting the motor repertoire required for different grasp actions (Rizzolatti et al., 2002). When our subjects were presented with an object, it is plausible that PMv neurons corresponding to the appropriate grasp would become differentially active. Hence, the cortico-cortical effects evoked by the conditioning pulse over PMv would reflect the active cell population, yielding a net facilitation of the muscle representation in M1 consistent with the upcoming grasp. In a previous study, we showed that, in the resting state, the net interactions between PMv and M1 are inhibitory (Davare et al., 2008). We assume that this resting inhibitory effect is cancelled out during the preparation of grasp due to the strong activation of a grasp-specific cell population in PMv with excitatory influences over M1 and leading to a net facilitation of M1 outputs to the appropriate muscle representation. In line with this view, we previously showed that the resting inhibitory influence from PMv to the 1DI muscle representation in M1 changed to facilitation during preparation for PG, a posture in which the 1DI muscle plays a key role (Davare et al., 2008).

Recent studies investigating the cortico-cortical connections in M1 reported a similar muscle-specific facilitation during preparation for a particular grasp (Cattaneo et al., 2005; Prabhu et al., 2007). In addition to many intrinsic connections, M1 receives a rich pattern of cortico-cortical inputs from non-primary motor areas, including the dorsal premotor cortex (PMd), the supplementary motor area (SMA) and the posterior parietal cortex (PPC) which probably convey information about selection of the motor command needed for an appropriate grasp (Civardi et al., 2001; Koch et al., 2006, 2007; O'Shea et al., 2007; Schluter et al., 1999; Bestmann et al., 2008). A major part of the cortico-cortical input to M1 originates from PMv (Prabhu et al., 2007; Cattaneo et al., 2005; Schmidlin et al., 2008; Shimazu et al., 2004; Dum and Strick, 2005), in line with functional imaging studies reporting PMv activation during grasping movements (Binkofski et al., 1999; Grezes et al., 2003).

Interestingly, for each muscle, we found a correlation between the amount of MEP facilitation and the difference of EMG activity among the two grasps. This correlation was significant both for the MEPs recorded while testing the PMv–M1 interactions and for those evoked by the paired-pulse TMS paradigm over M1. The latter corroborates the results of Cattaneo et al. (2005). However, we found that the amount of MEP facilitation was larger when conditioning PMv (PMv–M1) compared to conditioning M1 (M1_PP_). Hence, the regression slope was steeper when testing the M1 cortico-cortical inputs than the PMv–M1 interactions. In other words, a smaller difference in the facilitation of MEPs in the M1_PP_ condition compared with the PMv–M1 condition was associated with the same difference in voluntary EMG activity during grasp. It is likely that, compared to the PMv–M1 interactions, the cortical circuit probed by the paired-pulse paradigm (M1_PP_) assesses the transformation of grasp-related visuomotor information at a distinct, downstream, level in the cortical pathway, which could include inputs from PMd and SMA. Functional imaging studies report that grasping movements not only activate PMv but PMd and SMA as well (Ehrsson et al., 2001; Kuhtz-Buschbeck et al., 2001). Therefore, the grasp-related information issued from PMv, together with ‘movement selection’ information originating from PMd and SMA, could be combined within M1, giving a higher gain to the visuomotor information required to generate the proper motor command.

The present study demonstrates that TMS is able to reveal specific grasp-related populations of cells in PMv that have a net facilitatory influence over M1 outputs to hand muscles. This contrasts with a net inhibitory effect at rest (Davare et al., 2008) which is presumably overwhelmed by activation of neurons representing a specific grasping action in PMv. Altogether our results suggest that different neuronal populations in PMv, representing different grasping actions (Rizzolatti et al., 2002), have strong facilitatory influences over the hand muscle representations in M1 that need to be recruited during grasp. PMv could therefore act as a ‘conductor’, governing the gain and balance between the different grasp-related cells and generating the relevant information for M1 to emit a specific motor command for a particular grasp.
