# Supplementary material for: Ventral premotor to primary motor cortical interactions during object-driven grasp in humans
Source: Cortex. 2009 Oct;45(9):1050–7. doi: 10.1016/j.cortex.2009.02.011 (PMC2730595; doi:10.1016/j.cortex.2009.02.011)
Supplement: Supplementary file 1 [file mmc1.doc]

**Stimulation sites**

The coil position was precisely determined, in every subject, by means of a coregistration technique of the stimulation sites onto individual anatomical magnetic resonance images previously gathered for each subject (Brainsight, Rogue, Montreal). In order to target PMv, the coil was positioned over the caudal portion of the pars opercularis of the inferior frontal gyrus. In the present study, the mean normalized MNI coordinates of PMv were -55±2, 12±6, 17±7 mm (x, y, z, mean±SD; n=7), close to those reported by functional imaging studies (Binkofski et al., 1999; Ehrsson et al., 2001; Kuhtz-Buschbeck et al., 2001). Additionally, we have shown that a virtual lesion of this region impairs precision grasping (Davare et al., 2006) and is functionally connected to M1 (Davare et al., 2008). In order to target M1, the coil was positioned over the site where TMS induced the largest MEPs in both the 1DI and ADM muscle. The coregistration procedure confirmed that the M1 site overlapped the hand knob (Yousry et al., 1997); its mean normalized MNI coordinates were -35±4, -24±3, 58±11 mm (x, y, z, mean±SD; n=11), which are also comparable to those reported in functional imaging studies (Fink et al., 1997; Picard and Strick, 2001).

The mean Euclidian distance between PMv and M1 stimulation sites was 61±8 mm (mean±SD; n=11), a distance sufficient to allow positioning of both coils over the same hemisphere.

**References**

Binkofski F, Buccino G, Posse S, Seitz RJ, Rizzolatti G, and Freund H. A fronto-parietal circuit for object manipulation in man: Evidence from an fmri-study. *Eur J Neurosci,* 11: 3276-86, 1999.

Davare M, Andres M, Cosnard G, Thonnard JL, and Olivier E. Dissociating the role of ventral and dorsal premotor cortex in precision grasping. *J Neurosci,* 26: 2260-8, 2006.

Davare M, Lemon R, and Olivier E. Selective modulation of interactions between ventral premotor cortex and primary motor cortex during precision grasping in humans. *J Physiol,* 586: 2735-42, 2008.

Ehrsson HH, Fagergren E, and Forssberg H. Differential fronto-parietal activation depending on force used in a precision grip task: An fmri study. *J Neurophysiol,* 85: 2613-23, 2001.

Fink GR, Frackowiak RS, Pietrzyk U, and Passingham RE. Multiple nonprimary motor areas in the human cortex. *J Neurophysiol,* 77: 2164-74, 1997.

Kuhtz-Buschbeck JP, Ehrsson HH, and Forssberg H. Human brain activity in the control of fine static precision grip forces: An fmri study. *Eur J Neurosci,* 14: 382-90, 2001.

Picard N and Strick PL. Imaging the premotor areas. *Curr Opin Neurobiol,* 11: 663-72, 2001.

Yousry TA, Schmid UD, Alkadhi H, Schmidt D, Peraud A, Buettner A, and Winkler P. Localization of the motor hand area to a knob on the precentral gyrus. A new landmark. *Brain,* 120 ( Pt 1): 141-57, 1997.
